# Overconnectivity of the right Heschl's and inferior temporal gyrus correlates with symptom severity in preschoolers with autism spectrum disorder

**DOI:** 10.1002/aur.2609

**Published:** 2021-09-16

**Authors:** Daegyeom Kim, Joo Young Lee, Byeong Chang Jeong, Ja‐Hye Ahn, Johanna Inhyang Kim, Eun Soo Lee, Hyuna Kim, Hyun Ju Lee, Cheol E. Han

**Affiliations:** ^1^ Department of Electronics and Information Engineering Korea University Sejong Republic of Korea; ^2^ Clinical Research Institute of Developmental Medicine Seoul Hanyang University Hospital Seoul Republic of Korea; ^3^ Interdisciplinary Graduate Program for Artificial Intelligence Smart Convergence Technology Korea University Sejong Republic of Korea; ^4^ Department of Pediatrics, Hanyang University Hospital Hanyang University College of Medicine Seoul Republic of Korea; ^5^ Department of Child and Adolescent Psychiatry, Department of Psychiatry Seoul Hanyang University Hospital Seoul Republic of Korea; ^6^ Department of Molecular and Cellular Biology Harvard University Cambridge Massachusetts USA; ^7^ Department of Child Psychotherapy Hanyang University Graduate School of Medicine Seoul Republic of Korea

**Keywords:** autism spectrum disorder, brain networks, diffusion tensor imaging, graph theory, Heschl's gyrus, overconnectivity, preschool children

## Abstract

Previous studies have reported varying findings regarding the association of brain connectivity in autism spectrum disorder (ASD) with overconnectivity, underconnectivity, or both. Despite the emerging understanding that ASD is a developmental disconnection syndrome, very little is known about structural brain networks in preschool‐aged children with low‐functioning ASD. We aimed to investigate the structural brain connectivity of low‐functioning ASD using diffusion magnetic resonance imaging and graph theory to examine alterations in different brain network topologies and identify any correlations with the clinical severity of ASD in preschool‐aged children. Fifty‐two preschool‐aged children (28 with ASD and 24 with typical development) were included in the analysis. Graph‐based network analysis was performed to examine the global and local structural brain networks. Nodal network measures exhibited increased nodal strength in the right Heschl's gyrus, which was positively associated with all autistic clinical symptoms (Autism Diagnostic Observation Schedule and Childhood Autism Rating Scale [CARS]). The nodal strength of the right inferior temporal gyrus showed a moderate correlation with the CARS score. Using network‐based statistics, we identified a subnetwork with increased connections encompassing the right Heschl's gyrus and the right inferior temporal gyrus in preschool‐aged children with ASD. The asymmetric value in the inferior temporal gyrus exhibited right dominance of nodal strength in children with ASD compared to that in typically developing children. Our findings support the theory of aberrant brain growth and overconnectivity as the underlying mechanism of ASD and provides new insights into potential regional biomarkers that can detect low‐functioning ASD in preschool‐aged children.

**Lay Summary:**

This study supports the theory of aberrant brain growth and overconnectivity as an explanation for ASD. Measuring the right HG and inferior temporal gyrus provides new insights of potential regional biomarkers underpinning ASD in preschool‐aged children.

## INTRODUCTION

Autism spectrum disorder (ASD) is a neurodevelopmental disorder characterized by impaired social interactions, restricted communication skills, and unusually repetitive behaviors. The latest survey by the Center for Disease Control and Prevention reported that ASD has become increasingly prevalent over the past 15 years, with one in 54 children being diagnosed with this condition at present (Maenner et al., 2020). A major limitation when identifying ASD in preschool‐aged children is that the disorder is diagnosed mainly based on a child's developmental history and several psychological assessments at an early age. Due to the methodological heterogeneity in diagnosing ASD and inherent subjectivity among psychological assessments, interest has shifted to investigations of altered neuroconnectivity during early brain development (Randall et al., 2018). Consequently, identifying neuroimaging biomarkers has been a major focus of ASD research over the last decade to enable objective ASD diagnosis in young children and to elucidate the underlying neuropathological mechanism.

Despite neuroimaging advances and the emerging understanding of ASD as a disorder of developmental connectivity, limitations have been observed in the clinical management of preschool‐aged children with low‐functioning ASD because most studies regarding the developmental trajectory of brain connectivity have been conducted in high‐functioning school‐aged children. Of note, a heterogeneous ASD population with low‐ and high‐functioning subjects impedes our understanding of the underlying mechanisms of ASD, in terms of structural connectivity (overconnectivity or underconnectivity compared to that in typically developing individuals). Although failure to develop cognitive skills followed by normal verbal performance is an early warning sign of autism during early infancy, connectivity in low‐functioning ASD has not yet been characterized during early brain development. Studies focusing on the neurodevelopment of individuals with low‐functioning ASD have been very limited, but recent functional magnetic resonance imaging (fMRI) studies have suggested a distinctive neurodevelopmental profile in these individuals compared to that in individuals with high‐functioning ASD (Gabrielsen et al., 2018; Reiter et al., 2019). Although resting‐state fMRI studies have demonstrated hyper‐ or hypo‐connectivity of other cortical regions in low‐functioning ASD, there has been relatively little research into the existence of atypicalities in structural brain MRI of preschool‐aged children. Furthermore, few fMRI studies have shown differences in brain connectivity between low‐ and high‐functioning ASD; therefore, information about the relationship between the functioning level or symptom severity and altered connectivity requires further study.

A recent longitudinal study of 429 children aged 2–13 years reported that ASD boys with disproportionate megalencephaly exhibited early cerebral enlargement and found no evidence of cerebral brain volume regressions in ASD through late childhood (Lee et al., 2020). Tract‐based spatial statistics (Andrews et al., 2019; Blanken et al., 2017; Cheng et al., 2010; Weinstein et al., 2011) and region‐of‐interest (ROI)‐based analysis (Andrews et al., 2021; Ben Bashat et al., 2007; Solso et al., 2016; Temur et al., 2019) have demonstrated early accelerated brain growth in various brain regions in preschool‐aged children with ASD. Weinstein et al. reported a significant increase in fractional anisotropy (FA) in the left cingulum as well as the midbody of the corpus callosum in children with ASD between the ages of 2 and 6 years, supporting the view that the atypical trajectory of brain development results from early white matter overconnectivity (Weinstein et al., 2011). Bashat et al. showed that, compared to typically developing children (TDC), children with ASD between the ages of 1.8 and 3.3 years exhibited elevated FA values predominantly in the left frontal lobe, suggesting an accelerated maturation of white matter and early brain overgrowth (Ben Bashat et al., 2007). A meta‐analysis of diffusion tensor imaging (DTI) studies identified a decrease in FA values and increase in mean diffusivity (MD) in the corpus callosum, an essential structure for interhemispheric communication, in individuals with ASD as compared with TDC (Aoki et al., 2013; Di et al., 2018).

Recent studies considering changes in the developmental trajectory may account for the inconsistent results regarding age‐dependent or region‐specific abnormal brain connectivity, underlying how altered white matter integrity across early childhood in individuals with ASD is related to behavioral impairment. For example, a longitudinal study of 125 children with ASD between 2.5 and 7 years of age showed that the FA values in several brain regions of the splenium of the corpus callosum, superior longitudinal fasciculus, internal capsule, and cingulum initially increased, but later decreased, contributing to a slower developmental trajectory compared with TDC (Andrews et al., 2021). Solso et al. discovered age‐related changes in brain connectivity in the frontal fiber tracts of children with ASD, with abnormally higher FA suggesting local axonal overconnectivity in the first two years of life but slightly lower FA between the ages of 3 and 4 years suggesting under‐connectivity (Solso et al., 2016).

While previous DTI studies focused on microstructural changes, graph theoretic analyses of DTI data have been widely employed in various diseases since 2010. This approach models the brain as a complex network, where connectivity between brain regions is estimated from DTI data using a computational algorithm called tractography (Bullmore & Sporns, 2009; Mori & Barker, 1999). This approach facilitates the investigation of abnormalities in the connectivity and interaction between brain regions due to neural diseases (Bullmore & Sporns, 2009; Mori & Barker, 1999). In particular, network topological measures capture various aspects of interactions and are sensitive to the deterioration of brain networks. This approach may overcome the limitations of previous approaches, including volumetric analyses of structural MRI and microstructural changes of diffusion MRIs, and may complement them by providing insights into abnormalities not only in terms of local structural changes in single brain regions or tissue, but also in the global interactions between brain regions. Prior research on network connectivity using graph theory in preschool‐aged children with ASD is scarce, with most studies focusing on functional connectivity rather than on structural connectivity in children with high‐functioning ASD. Different aspects and modalities between structural and functional MRI could represent a critical gap in our understanding of brain function across the autism spectrum. Thus, in this study, we focused on low‐functioning ASD, using structural MRI, and highlighted a functional relationship between altered connectivity (based on graph theory) and symptom severity in individuals with low‐functioning ASD.

To date, only six studies that used the graph theory have explored the structural network connectivity in preschool‐aged children with ASD (Billeci et al., 2019; Carpenter et al., 2019; Lewis et al., 2014; Li et al., 2018; Qian et al., 2018; Qin et al., 2018) (Supplementary Table 1). To the best of our knowledge, no previous study has used the graph theory to explore the different patterns of brain networks in a low‐functioning ASD cohort with a mean age below 6 years. Based on structural connectivity MRI and graph theory, we aimed to investigate different topological brain networks that are associated with symptom severity as potential neuroimaging biomarkers between preschool‐aged children with low‐functioning ASD and TDC.

## MATERIALS AND METHODS

### Subjects

Participants were infants aged 3–6 years who had been diagnosed with ASD or were TDC at Hanyang University Medical Center between January 2017 and November 2019. Infants who had been diagnosed with ASD at the Child and Adolescent Psychiatry Department at Hanyang University Medical Center were recruited to the Hanyang Inclusive Clinic for Developmental Disorders at Hanyang University Seoul Hospital. Infants with ASD underwent intellectual quotient (IQ) assessment and Korean Social Maturity Scale (K‐SMS) evaluation as part of their clinical assessment at the Hanyang Inclusive Clinic for Developmental Disorders. Thus, our study participants with low‐functioning ASD, referred to as preschool‐aged children with ASD and IQs below 70, were enrolled as part of the Medicine·Engineering·Bio Research Project in Hanyang Inclusive Clinic for Developmental Disorders. Subjects with low‐functioning ASD who met the cut‐off points for autism on the Autism Diagnostic Observation Schedule (ADOS) and Childhood Autism Rating Scale (CARS) and low‐function IQ levels on the Wechsler Preschool and Primary Scale of Intelligence IV (WPPSI‐IV) were included in this study. The exclusion criteria for participants with ASD were as follows: family history of ASD, known genetic disorder, history of or current brain trauma, structural brain abnormalities, seizure, other neurological disorders, or any psychiatric disorder.

The diagnosis of ASD was confirmed at the Child and Adolescent Psychiatry Outpatient Clinic at Hanyang University Hospital using the ADOS, CARS, and expert clinical diagnosis by a pediatric psychologist according to the criteria of the Diagnostic and Statistical Manual of Mental Disorders, Fourth Edition (Kaufman et al., 1997; Kim et al., 2004). The ADOS is a semi‐structured observational tool that scores two independent subdomains of autistic symptoms: social affective (SA) and restrictive repetitive behavior (RRB). It is currently considered the gold standard for diagnosing ASD (Lord et al., 2000). The examiner administered one of the four modules according to the participant's language level and age (ranging from nonverbal young children to verbally fluent adults). IQ levels were determined using the WPPSI‐IV, a commonly used test of cognitive development for preschool children aged 30–56 months (Wechsler, 2012), administered by trained psychologists (Wechsler et al., 2019).

The TDC group included children with no signs of developmental delay. Developmental ability was screened in all infants at a routine health check‐up using the Korean Developmental Screening Test for Infants and Children at Hanyang University Medical Center Pediatrics Department. This screening tool is composed of six subscales: motor function, fine motor function, cognition, language, social interaction, and self‐help and was categorized as screen‐positive when any one of the domains was <−2 standard deviations (SD) from an age‐appropriate level. TDC with developmental scores within 2 SDs of the mean on all subscales of the Korean Developmental Screening Test at the Hanyang Inclusive Clinic for Developmental Disorders were recruited in the TDC group. TDC who underwent a detailed neurological examination and developmental assessment by a pediatrician were enrolled in this study as a part of the Medicine·Engineering·Bio Research Project at Hanyang Inclusive Clinic for Developmental Disorders to obtain developmental measures, such as MRI examination for DTI, IQ, and CARS. Inclusion criteria for the TDC group were no evidence of mental retardation, pervasive developmental disorder, specific language impairment, or any known developmental, neurological, or behavioral problems and normal imaging as judged by a clinical neuroradiologist. Individuals with developmental disabilities (CARS score >28) were also excluded from the TDC group. Those with an IQ <70 were also excluded.

Seventy‐one participants aged 3–6 years were recruited from Hanyang University Medical Center. This included 39 individuals who had been diagnosed with ASD and 32 TDC. Nine patients with ASD and six TDC were subsequently excluded due to poor imaging quality, and four patients with ASD were excluded due to missing IQ data. In total, 52 preschool‐aged children (male/female; 37/15), including 28 with ASD and 24 TDC were included in the analysis. Basic clinical data, including gestational age (GA), birth weight, sex, age at imaging, medication history, and IQ were prospectively recorded for all participants using clinical trial forms in the hospital.

The Institutional Review Board of Hanyang University Seoul Hospital (No. 2017‐04‐004) approved the study protocol and scanning procedures. This study was conducted in accordance with the principles of the Declaration of Helsinki. Written informed consent was obtained from all parents/guardians, and the children provided verbal consent to participate after receiving an explanation of the study prior to enrollment.

### MRI scanning procedure

We used sedation to obtain MRI scans of all preschool‐aged children with ASD and all TDC. However, obtaining MRI scans of preschool‐aged children poses many methodological challenges, such as head motion and loud acoustic noise. To avoid these issues, we focused on reducing head motion under sedation to acquire high‐quality images using earplugs, headphones, or both. MRI brain scans were taken ranging from 20 to 30 min, and all children underwent an MRI scan during their nap time. The sedation and details of the MRI procedure were explained to the parents. A documented review of the medical history and physical examination was performed by the physician. Sedation was administered by a trained pediatric nurse after an appropriate interval of fasting before sedation. Chloral hydrate was selected due to its low complication rates and high efficacy when used in accordance with the guidelines for children's sedation as published by the American Academy of Pediatrics (Committee on Drugs. American Academy of, 2002; Vade et al., 1995). All children sedated for the MRI scanning procedure were carefully monitored using pulse oximetry and were supervised by a skilled physician who was trained in providing pediatric advanced life support. The children's cardiorespiratory status and vital signs were recorded every 5 min. The imaging session was interrupted if the child moved or woke up during scanning. After the imaging procedure was completed, discharge was permitted only when sedation effects were discontinued and proper recovery had been achieved.

### MRI data acquisition

MRI brain scans were acquired using a 3.0‐T MRI scanner (Philips Real‐Time Compact Magnet 3.0‐T MRI system, Achieva 3.0‐T X‐series; Philips Healthcare, Best, The Netherlands), equipped with a 16‐channel SENSE head coil. T1‐weighted images, including sagittal and axial T1 turbo field echo sequences, were obtained with the following parameters: TR = 8.3 ms, TE = 4.6 ms, field‐of‐view (FoV) = 224 mm × 224 mm, spatial resolution = 0.6 mm × 0.6 mm × 1 mm, and slice thickness = 1 mm. T2‐weighted images were obtained to exclude white matter abnormalities. The turbo spin echo T2 scan imaging parameters were as follows: TR = 3000 ms, TE = 100 ms, FoV = 180 mm × 180 mm, spatial resolution = 0.5 mm × 0.5 mm × 4 mm, and slice thickness = 4.0 mm. Radiological evaluation was performed by an experienced pediatric neuroradiologist who was blinded to all other data. DTI was performed using a single‐shot spin‐echo planar sequence with a SENSE factor of 2 and echo planar imaging factor of 51 (TR = 8192 ms, TE = 76 ms, FoV = 224 mm × 224 mm, spatial resolution = 2 mm × 2 mm × 2 mm, and slice thickness = 2.0 mm). The slice orientation was axial and parallel to the anterior–posterior commissure line. Seventy‐four axial sections covered the entire hemisphere and brainstem. The diffusivities were measured along 15 directions using an electrostatic gradient model (*b* = 800).

### Image preprocessing and network construction

**FIGURE 1:**
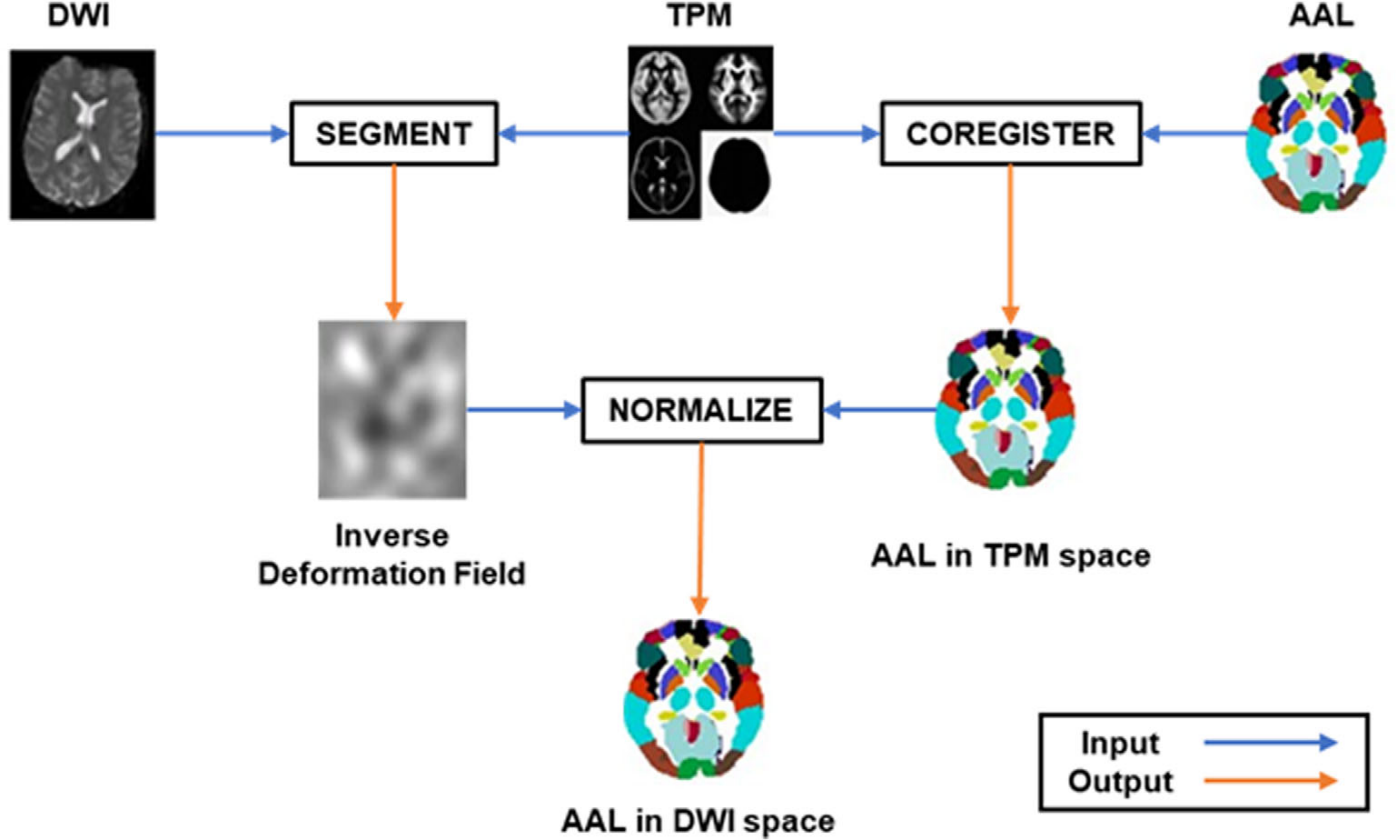
Schematic of the registration of AAL of the diffusion tensor imaging (DTI) space using SPM software. In each stage, blue lines indicate input data and orange lines indicate output data. We first segmented DWIs to obtain a deformation field to the tissue probability map (TPM) space. We also registered the AAL atlas to the TPM space. The AAL atlas co‐registered to the TPM space was normalized by applying the inverse of the deformation field obtained in the segmentation procedure. As a result, we obtained the registered AAL atlas in the DWI space for each individual

To define brain networks using processed MRI data, we defined nodes as the brain regions and edges as the number of streamlines between a pair of brain regions. To define the nodes, we registered images in standard space (MNI‐152), in which the automated anatomical labeling (AAL) atlas (Tzourio‐Mazoyer et al., 2002) resides on diffusion‐weighted images (DWIs), using statistical parametric mapping (SPM) 12 (Figure 1). We first segmented DWIs to obtain the deformation field in a tissue probability map (TPM) space. We used the average TPM template because the subjects were children. Because the segmentation procedure was carried out in the TPM space, prior to segmentation, SPM was performed for mutual information‐based affine registration and normalization of the DWI of each subject onto the TPM space, resulting in its deformation field. Second, we registered the AAL atlas to the TPM space. The AAL atlas co‐registered to the TPM space was then normalized by applying the inverse of the deformation fields obtained in the previous procedure, resulting in the AAL atlas within the individual's diffusion space. We matched the voxel size by reslicing the image with the original DWI. In the normalization and co‐registration procedure, we used the nearest‐neighborhood interpolation method, which led to clearer regional boundaries when converting the regions in the standard space into other spaces. Finally, we obtained the registered AAL atlas in the DWI space of each individual. We used 90 brain regions (78 cortical and 12 subcortical brain regions) as the nodes, excluding regions in the cerebellum.

To obtain streamline tractography from processed DWIs, deterministic tractography was performed. Because unwanted movements can occur during MRI scanning, we performed eddy‐current correction on the DWIs. Specifically, we adjusted unwanted movements by registering all volumes with non‐zero gradient directions to a reference volume without diffusion encoding, using the eddy‐current correction toolbox of the FMRIB's Diffusion Toolkit (v.3.0). We also rotated the gradient directions appropriately during the alignment procedure. We restricted the seed regions to white matter to avoid artifacts in tractography. The white matter mask was obtained using the FMRIB's Automated Segmentation Tool (FAST) (Zhang et al., 2001) on brain DWI processed using the Brain Extraction Tool (v.2.1) (Jenkinson et al., 2002). This was followed by using the Fiber Assignment by Continuous Tracking (FACT) algorithm, with a 45° angular threshold, through the diffusion toolkit along with TrackVis (Mori & Barker, 1999; Wang et al., 2007).

Finally, we obtained connectivity matrices of the brain network by counting the number of streamlines between any pair of two defined ROIs using the UCLA multimodal connectivity package (UMCP, http://ccn.ucla.edu/wiki/index.php). As a result, we obtained a 90 × 90 connectivity matrix.

### Network measures

We quantified the properties of brain networks using the Brain Connectivity Toolbox (Rubinov & Sporns, 2010). For the nodal properties of the networks, we measured the nodal degree and strength, clustering coefficient, local efficiency, and regional efficiency for each node. For the global properties of the networks, we measured the edge density, total strength, clustering coefficient, characteristic path length (CPL), small‐worldness, global efficiency, and local efficiency. This Toolbox supports MATLAB implementation for computing these measures (Rubinov & Sporns, 2010).

#### Nodal degree and edge density

The degree of a node is the number of neighboring nodes connected to the node. If the connection between the ith and jth nodes exists, $a_{\mathrm{ij}}$ = 1; otherwise, $a_{\mathrm{ij}}$ = 0. The degree of the ith node, *K*_*i*_, is defined by summing $a_{\mathrm{ij}}$, where *N* is the number of nodes [Equation (1)].

(1) $$K_{i}=\sum_{j\in N}a_{\mathrm{ij}}$$

The edge density of a network, ρ, is defined as the ratio of the number of connected edges to the number of all possible edges in a network. It is a counterpart of the nodal degree in the global measure [Equation (2)].

(2) $$\rho =\frac{1}{N\left(N-1\right)}\sum_{i\in N}K_{i}$$

#### Nodal strength and total strength

The nodal strength is the sum of all the edge weights connected to the node. The nodal strength of the ith node, $S_{i}$, is defined by summing *w*_*ij*_, where *w*_*ij*_ is the edge weight between the ith and jth nodes, and *N* is the number of nodes [Equation (3)].

(3) $$S_{i}=\sum_{J\in N}w_{\mathrm{ij}}$$

The total strength is defined by the summation of all edge weights in the whole network. Because our connectivity matrix is symmetric, the total strength is half of the summation.

#### Clustering coefficient at the nodal and global levels

The clustering coefficient measures how well nodes are locally clustered. The clustering coefficient at the nodal level is defined by the fraction of the number of triangles connected with a node to the number of possible triangles between all neighboring nodes. In a weighted network, weights should be considered together while counting the number of triangles; thus, the triangle intensity is employed (Onnela et al., 2005). The triangle intensity is d1efined as the cubic root of the product of the edge weights in a triangle. The total intensity of the ith node, $t_{i}$, is calculated by the summation of the triangle intensities of all the connected triangles [Equation (4)].

(4) $$t_{i}=\sum_{j,k\in N}{\left(w_{\mathrm{ij}}w_{\mathrm{jk}}w_{\mathrm{ki}}\right)}^{1/3}$$

where $w_{\mathrm{ij}}$, $w_{\mathrm{jk}}$, and $w_{\mathrm{ki}}$ represent the edge weights between two neighboring nodes when the jth and kth nodes are the neighboring nodes connected with the ith node, and *N* is the number of nodes. The number of possible triangles connected to the ith node is identical to selecting any two neighboring nodes connected to the node [$K_{i}\left(K_{i}-1\right)/2$, where $K_{i}$ is the degree of the node]. The clustering coefficient of the node is calculated as follows [Equation (5)].

(5) $$C_{i}=\frac{2}{K_{i}\left(K_{i}-1\right)}\sum_{j,k\in N}t_{i}$$

The clustering coefficient at the global level is defined as the average of the nodal clustering coefficient over all nodes.

#### Regional efficiency and CPL

The regional efficiency and CPL measure how well a node communicates with others. Better communication between two nodes can be measured through a shorter path length, where the path length is the geodesic distance between two nodes. Since the strong edge weight between two nodes represents better communication in our weighted network, we remapped each edge weight as its reciprocal to find the shortest path lengths through the Dijkstra algorithm. The two topological measures are different in the way of averaging the shortest path lengths over the whole network. For regional efficiency, we averaged their reciprocals [Equation (6)], while the CPL was defined as their average of the shortest path length [Equation (7)], where $d_{\mathrm{ij}}$ is the shortest path length between the ith and jth nodes, and D is the number of finite$\;d_{\mathrm{ij}}$.

(6) $$E_{i}=\frac{1}{N-1}\sum_{i\neq j\in N}\frac{1}{\mathrm{dij}}$$

(7) $$L=\frac{\sum_{d_{\mathrm{ij}\neq \infty }}d_{\mathrm{ij}}}{D}$$

#### Local efficiency at the nodal and global level

Similar to the clustering coefficient, the local efficiency is a measure of how well neighboring nodes, connected with a node, communicate between them. While the clustering coefficient focused on the number of edges and/or edge strength, the local efficiency also considers the shortest path lengths between neighboring nodes. Specifically, local efficiency is defined as the average efficiency of a local subgraph centered on a certain node. A local subgraph of the ith node consists of a set of neighboring nodes for the local efficiency of a weighted network at the nodal level. We calculated the total intensity combined with the shortest path length for all pairs of any two nodes in the local subgraph. In contrast to the total intensity of the clustering coefficient, the total intensity of the local efficiency is calculated by replacing $w_{\mathrm{jk}}$ with the reciprocal of the shortest path length ($d_{\mathrm{jk}}$) between the jth and kth nodes [Equation (8), numerator]. We then averaged these values [Equation (8)]. The local efficiency at the global level is defined as the average local efficiency at the nodal level.

(8) $$E_{\mathrm{loc},\text{nodal}}\left(i\right)=\frac{1}{2}\sum_{i\in N}\frac{\sum_{j,k\in N,j\neq i,k\neq i}{\left(w_{\mathrm{ij}}w_{\mathrm{ik}}{\left[d_{\mathrm{jk}}\right]}^{-1}\right)}^{1/3}}{K_{i}\left(K_{i}-1\right)}$$

#### Global efficiency

Global efficiency measures how well nodes in a network communicate with each other on average. We defined the global efficiency by the average of the reciprocal of the shortest path length between any pair of nodes, where *N* is the number of nodes [Equation (9)].

(9) $$E_{\text{glob}}=\frac{1}{N}\sum_{i\in N}\frac{\sum_{j\in N,j\neq i}d_{\mathrm{ij}}^{-1}}{N-1}$$

#### Small‐worldness

Small‐worldness characteristics capture a balance between high clustering and low CPL. As explained above, high clustering means that neighbors of a certain node are also densely connected; thus, information communication between them may also be good. On the other hand, a lower CPL indicates better communication at the global level. Thus, a network with small‐worldness characteristics has good information communication capabilities, not only locally, but also globally. Small‐worldness (σ) is computed as the ratio between the clustering coefficient and CPL [Equation (10)].

(10) $$\sigma =\frac{C}{L}$$

### Network‐based statistics

Because changes in the network topology can affect structural changes in the brain network, we investigated the changes in structural connectivity in the brain network using network‐based statistics (NBS) with a general linear model (GLM) (Zalesky et al., 2010). NBS with GLM extract subnetworks that consist of edges with significant group differences while controlling for the effect of covariates. We constructed a GLM model that provided the test statistics for each connection. By applying a certain threshold for the test statistics for all edges, the connections exceeding the threshold formed topological clusters that were connected to the graph components. NBS estimates the significance level of whether each cluster occurs higher than random chance. NBS uses permutation testing for this estimation, as summarized below. First, we randomly permuted the group information and performed a GLM analysis for each connection with the permuted group information. Then, using the same threshold for the original group assignment, we thresholded the connections and formed topological clusters. The size of the largest component was collected for permuted group information. Repeating this procedure several times (10,000 times in this experiment), we obtained an empirical null distribution of the size of the largest component. Finally, we computed the family‐wise error rate for each component of the original group assignment by simply counting the number of occurrences of randomly formed clusters that were larger than the designated cluster.

### Asymmetry index

We investigated the asymmetric properties of brain regions using the asymmetry index (AI). The AI quantifies the hemispheric imbalance of a certain topological parameter. It is defined by $\left(R_{\text{measure}}-L_{\text{measure}}\right)/\left(R_{\text{measure}}+L_{\text{measure}}\right)$, where $R_{\text{measure}}$ and $L_{\text{measure}}$ are topological measures of the right and left hemispheres, respectively. Positive AI values represent a rightward bias of the topological measure, while negative AI values represent a leftward bias.

### Statistical analysis

We compared various network measures between groups using permutation‐based analysis of covariance (ANCOVA) (Cho et al., 2018; Genovese et al., 2002; Nichols et al., 2008), controlling for the effects of GA, sex, and age at imaging. The permutation test is a method that tests the null hypothesis that different groups come from the same distribution. We obtained the null distribution by resampling the data sets *N‐1* times by random assignment of all subjects into one of two groups and computing the test statistics (*F*) in each permuted dataset through ANCOVA. To estimate the significance level of group differences, we first computed the *F*‐value for the original dataset. Then, we defined the significance level by the fraction of occurrences whose *F*‐values of the resampled data sets were not less than the *F*‐value of the original dataset. To compute the test statistics, we used our in‐house codes and the LinStat library (2006b) (Mulchrone, 2003). We used 10,000 permutations as *N*. The seven global measures were correlated with clinical severity scores using partial correlation with p‐values corrected using a false discovery rate (FDR) procedure. For the nodal measures, we performed the FDR procedure on more than 90 nodes as multiple comparison corrections (Benjamini & Cohen, 2017; Benjamini & Hochberg, 1995).

To investigate how changes in the network topology affected ASD symptoms, we performed a correlation analysis between network measures that had group differences and autistic clinical symptoms (based on the CARS and ADOS) in the ASD group. We used a partial correlation coefficient to control for the effects of GA, sex, and age at imaging in the ASD group. We tested whether AI values were significantly different from zero using a Wilcoxon signed‐rank test and compared them between groups using rank‐sum tests as the data did not follow a normal distribution. A Kolmogorov–Smirnov test was performed to test for normality. In addition, we investigated the relationship between AI and autistic clinical symptoms (based on the CARS and ADOS) using partial correlation analysis, while controlling for the effects of GA, sex, and age at imaging.

## RESULTS

There were no significant differences in age between the low‐functioning ASD and TDC groups. The low‐functioning ASD group demonstrated significantly higher CARS scores and lower IQ scores than the TDC group (Table 1). The IQ range of the ASD group was 40–67, while that of the TDC group was 75–111.

**TABLE 1:** Clinical characteristics

	TDC (*n* = 24)	ASD (*n* = 28)	*p*‐value^a^
Age (months)	46.6667 ± 14.0702	43.3214 ± 9.2017	0.4744^a^
GA (weeks)	38.2500 ± 2.4539	36.4643 ± 3.7167	0.0502^a^
Sex (Male/Female)	18/6	19/9	0.5709^b^
FSIQ	87.2857 ± 14.4777	46.5200 ± 9.4742	<0.001^a^
CARS	19.5556 ± 3.2156	33.9167 ± 5.0469	<0.001^a^
SMS		58.4776 ± 9.2657	
ADOS (total)		19.3333 ± 3.6240	
ADOS (SA)		16.7619 ± 2.6815	
ADOS (RRB)		2.5714 ± 1.7485	

*Note*: Data are presented as the mean ± standard deviation.

^a^ *P*‐values from Student's *t*‐test.

^b^ *P*‐values from the chi‐square test.

Abbreviations: TDC, typically developing children; ASD, autism spectrum disorder; FSIQ, full scale intelligence quotient; CARS, childhood autism rating scale; SMS, social maturity scale; ADOS, autism diagnostic observation schedule; SA, social affective; RRB, restrictive repetitive behavior.

### Group differences in network topological measures

We first compared the global properties of the brain structural network between the low‐functioning ASD and TDC groups, correcting for GA, sex, and age at imaging. There was no significant difference in the global measures between the low‐functioning ASD and TDC groups. (Supplementary Table 2).

**FIGURE 2:**
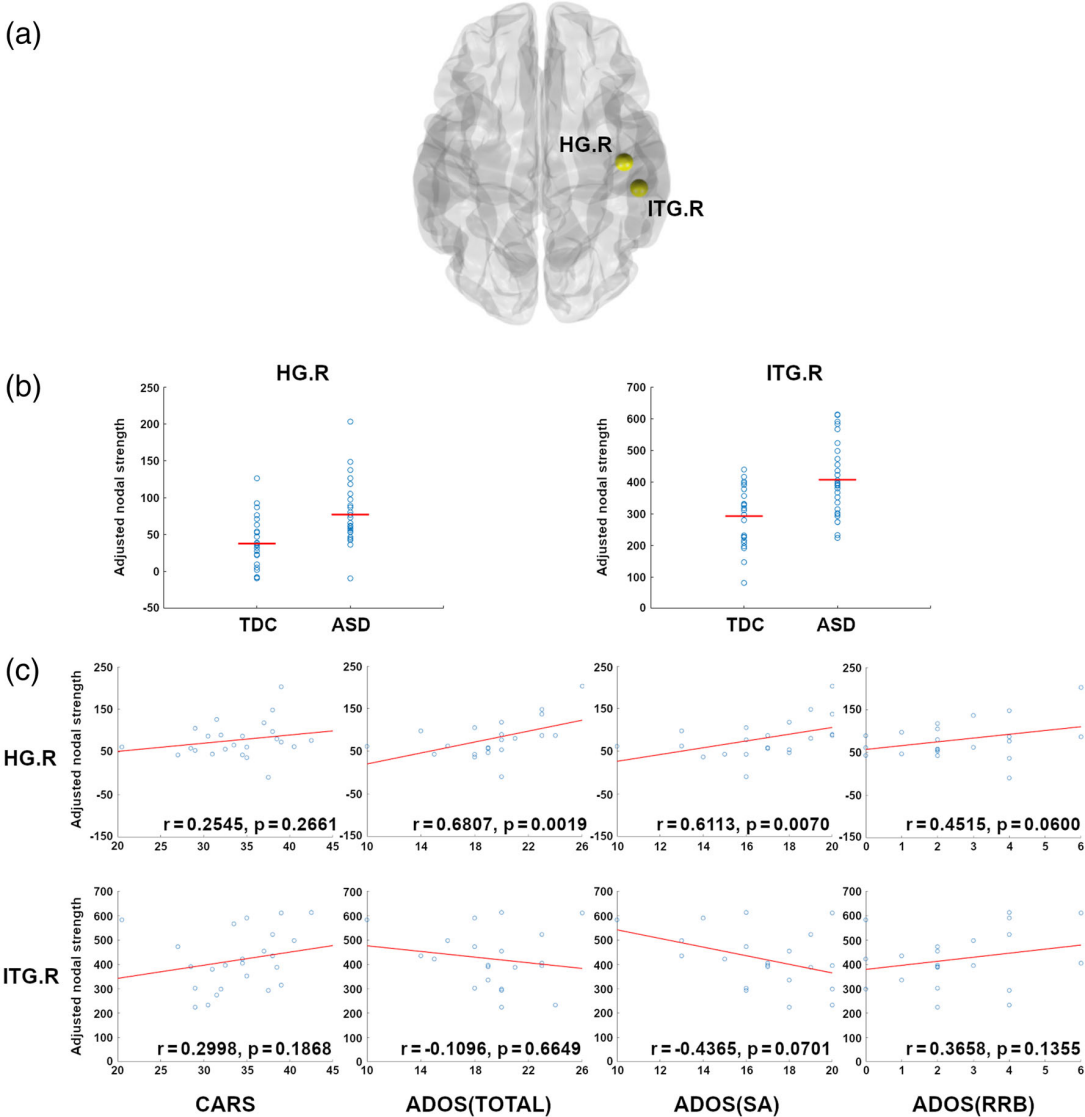
Group differences for the nodal measures and their correlations with autism spectrum disorder (ASD) symptoms. (a) Brain regions with stronger nodal strength in the ASD group compared to in TDC were marked as yellow circles in a transverse view of both hemispheres. (b) the nodal strength in each group was shown with scatter plots. Blue circles represent each subject, and red lines indicate the group average. (c) the nodal strength was correlated with ASD symptoms (partial correlation coefficients in‐set), where blue circles represent each subject and red lines are linear fitting lines. HG, Heschl's gyrus; ITG, inferior temporal gyrus

The nodal topological measures were compared between the low‐functioning ASD and TDC groups. We found that the nodal strength of the right Heschl's gyrus (HG; FDR‐adjusted *p*‐value: 0.0315, in TDC: 168.1250 ± 37.3099, in ASD: 201.3571 ± 42.2473) and right inferior temporal gyrus (ITG; FDR‐adjusted *p*‐value: 0.0315, in TDC: 679.0417 ± 94.0673, ASD: 774.8571 ± 119.7602) were higher in the ASD group (Figure 2(a)‐(b) and Table 2).

**TABLE 2:** Differences in nodal strength between children with ASD and TDC and their correlations with ASD symptoms

	Group difference			Correlation with ASD symptoms		
Nodes	TDC	ASD	*p*‐value^a^	Scores	*r*‐value^b^	*p*‐value^b^
Right Heschl's gyrus	168.1250 ± 37.3099	201.3571 ± 42.2473	0.0315	CARS	0.2545	0.2661
	–	–	–	ADOS (TOTAL)	0.6807	0.0019
	–	–	–	ADOS (SA)	0.6113	0.0070
	–	–	–	ADOS (RRB)	0.4515	0.0600
						
Right inferior temporal gyrus	679.0417 ± 94.0673	774.8571 ± 119.7602	0.0315	CARS	0.2998	0.1868
	–	–	–	ADOS (TOTAL)	−0.1096	0.6649
	–	–	–	ADOS (SA)	−0.4365	0.0701
	–	–	–	ADOS (RRB)	0.3658	0.1355

*Note*: Data are presented as the mean ± standard deviation.

^a^ *P*‐values from permutation‐based ANCOVA controlling for gestational age, sex, and age at imaging.

^b^ Partial correlation coefficient and *P*‐value, controlling for gestational age, sex and age at imaging.

Abbreviations: ASD, autism spectrum disorder; TDC, typically developing children; ADOS, autism diagnostic observation schedule; SA, social affective; RRB, restrictive repetitive behavior; CARS, childhood autism rating scale.

### Correlations with ASD symptoms

We analyzed the correlation between network measures and autistic clinical symptoms with the CARS total score and scores of all ADOS subdomains (SA, RRB, and total, respectively). There was no significant correlation between the global measures and symptoms of the ASD group. (Supplementary Table 3).

The nodal strength of the right HG was associated with all autistic clinical symptoms in the ASD group [total ADOS score (*r* = 0.6807, *p* = 0.0019) and ADOS SA score (*r* = 0.6113, p = 0.0070); Figure 2(c) and Table 2)].

### Increased connectivity in the ASD group

**FIGURE 3:**
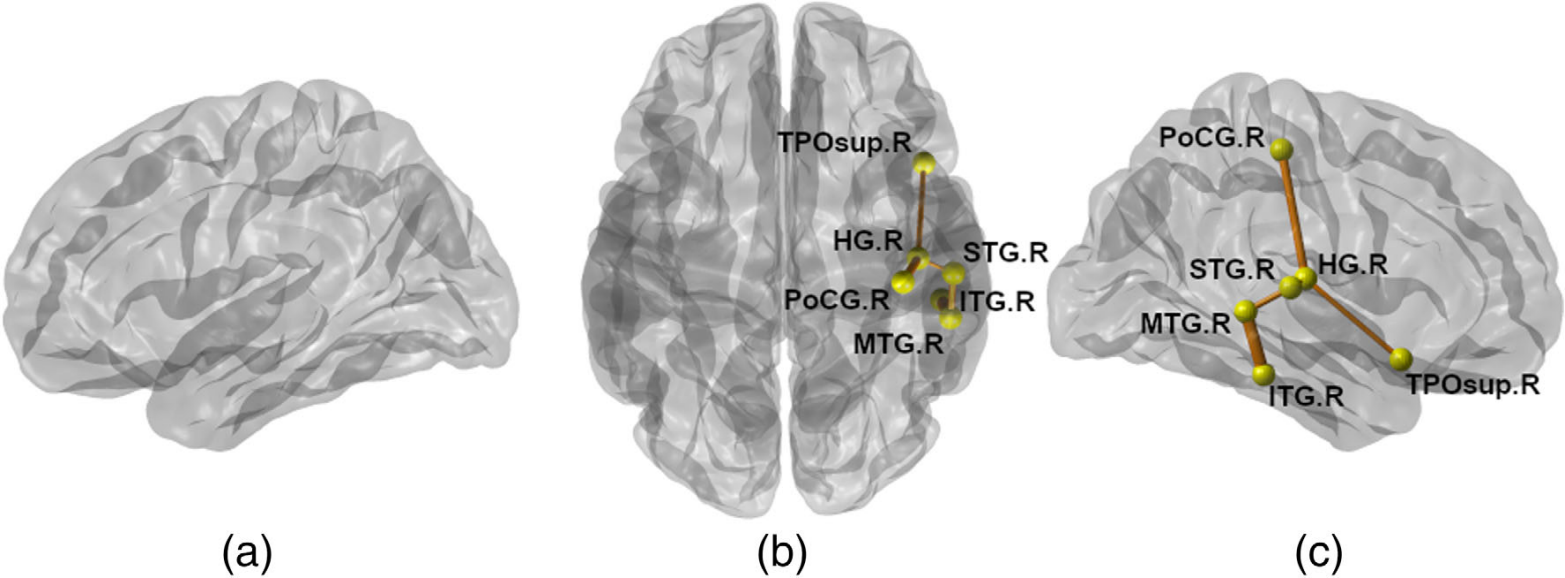
Abnormal subnetwork identified through NBS. Lateral view of the left hemisphere (a); transverse view of both hemispheres (b); and lateral view of the right hemisphere (c). Yellow circles indicate brain regions, and orange lines indicate the connections between them. The subnetwork consists of five nodes and four connections, and all connections were stronger in children with autism spectrum disorder than in TDC. HG, Heschl's gyrus; TPOsup, superior temporal pole gyrus; PoCG, postcentral gyrus; STG, superior temporal gyrus; ITG, inferior temporal gyrus; MTG, middle temporal gyrus

Using the NBS, we identified a subnetwork that had increased connectivity in the low‐functioning ASD group as compared to the TDC group (corrected *p* = 0.0327), while controlling for GA, sex, and age at imaging. The extracted subnetworks were identical, with threshold values between 2.3 and 2.4. Here, we report on the subnetworks with the largest threshold value. The identified subnetwork consisted of the following connections: connection between the right superior temporal gyrus and right middle temporal gyrus, right ITG and right middle temporal gyrus, right HG, and right postcentral gyrus. All edges identified resided in the right hemisphere and were stronger in the ASD group than in the TDC group (Figure 3 and Table 3).

**TABLE 3:** Structural connectivity within subnetworks identified through NBS

Connections	TDC	ASD	T‐Statistics^a^
STG.R – MTG.R	381.5417 ± 103.3504	460.7500 ± 108.0475	2.7384
ITG.R – MTG.R	263.0417 ± 48.3092	321.5714 ± 64.1869	3.4681
HG.R – STG.R	48.5750 ± 15.6297	63.5357 ± 24.4790	2.4251
HG.R – TPOsup.R	1.7083 ± 2.9997	4.6071 ± 4.5570	2.6610
HG.R – PoCG.R	0.8333 ± 1.8337	5.0357 ± 7.1049	3.0261

*Note*: Data are presented as the mean ± standard deviation.

^a^ T‐statistics of group differences for connections identified by network‐based statistics.

Abbreviations: STG, superior temporal gyrus; MTG, middle temporal gyrus; ITG, inferior temporal gyrus; HG, Heschl's gyrus; TPOsup, superior temporal pole gyrus; PoCG, postcentral gyrus.

### Asymmetry of nodal strength in the ASD group

As we found an abnormality in the nodal strength of HG and ITG, we investigated their AI and compared the asymmetry measure between the two groups. We found a rightward asymmetry in the nodal strength of HG (in TDC: 0.1067, in ASD: 0.1774, Supplementary Table 4), but the difference was not statistically significant (*p* = 0.0551). In the ITG, the TDC group exhibited leftward asymmetry in nodal strength, while children with ASD showed rightward lateralization in nodal strength (in TDC: −0.0540, in ASD: 0.0226). Consequently, we found a significant difference between groups in the AI values of the ITG (rank‐sum test, *p* < 0.0072), representing right hemispheric lateralization in children with ASD.

## DISCUSSION

### Global network characteristics of low‐functioning ASD

This study showed no significant difference in global topological measures between preschool‐aged children with low‐functioning ASD and TDC. In another study, there were significant reductions in global and local efficiency bilaterally in 24‐month‐old infants diagnosed with ASD compared to infants not classified as ASD, supporting the underconnectivity theory of ASD (Lewis et al., 2014). Another study also showed a significant decrease in global efficiency in both binary and density‐weighted networks, which may indicate reduced integration, in adult males with ASD compared to typically developed adults (Roine et al., 2015). In contrast, a previous study examining the graph metrics of functional networks reported that adolescents with ASD had a higher global efficiency and lower local efficiency than TDC (Rudie et al., 2012). There was no significant correlation between global network measures and clinical symptoms of ASD in the present study. However, a previous study stated that overconnectivity is seen in the brains of preschool‐aged children with ASD (Qin et al., 2018), while other studies provided evidence that underconnectivity is associated with ASD in school‐aged children (Brito et al., 2009; Payabvash et al., 2019) with decreased CPL, increased global efficiency, and an increased clustering coefficient compared with TDC (Li et al., 2018).

Of note, in children with ASD, the developmental period under the age of 6 years is a crucial phase of neural network formation that is critical for early intervention. Although low‐functioning ASD in preschool‐aged children is not completely identifiable in neuroimaging studies, low‐functioning ASD in the presence of cognitive impairment is more likely to develop as early as the second year of life in children with the core features of ASD. A recent study by Gabrielsen et al. that targeted children with ASD and poor verbal and cognitive performance found decreased within‐network connectivity in default, salience, auditory, and frontoparietal networks and increased between‐network connectivity between the default and dorsal attention and frontoparietal networks compared to those in high‐functioning ASD (Gabrielsen et al., 2018). This suggests decreased network segmentation and integration in children with low‐functioning ASD (Gabrielsen et al., 2018). Another study reported functional underconnectivity within the default mode network and ventral visual stream, suggesting reduced network integration in individuals with low‐functioning ASD and reduced network segregation in those with high‐functioning ASD (Reiter et al., 2019).

### Altered nodal network in low‐functioning ASD and correlation with clinical severity

Atypical functional and structural brain connectivity has been studied in multiple brain regions and white matter tracks in individuals with ASD (Chien et al., 2015; Keown et al., 2013; Qin et al., 2018). We showed significant increases in nodal strength of the right HG and a marginal increase in the right ITG of children with ASD compared with TDC, which positively correlated with the severity of ASD. In addition, NBS identified a potentially disturbed subnetwork in the right hemisphere, which predominantly comprised temporo‐parietal connectivity in the ASD group (Figure 3 and Table 3) and positively correlated with the CARS total score and scores of all ADOS subdomains. The nodal overconnectivity in preschool‐aged children with ASD may be due to early brain overgrowth. In addition, reduced synaptic pruning and synapse elimination in the brain may be attributed to the clinical symptoms of ASD. Our findings are consistent with those of an earlier study by Keown et al. which used graph theory and showed increased local connection density in the temporo‐occipital regions in the ASD group which positively correlated with ADOS scores (Keown et al., 2013). Likewise, school‐aged boys with high‐functioning ASD have shown overconnectivity between the right posterior temporo‐parietal junction and right temporo‐occipital cortex compared to controls; this overconnectivity positively correlated with social deficits (Chien et al., 2015). In a DTI study of topological networks in preschool‐aged children, Qin et al. showed that the nodal efficiency of the left precuneus in the parietal lobe was higher in patients with ASD than in controls; this was positively associated with the severity of ASD symptoms (Qin et al., 2018). In contrast, another study of 24‐month‐old toddlers found significantly lower global and local efficiency in the temporal, parietal, and occipital lobes of infants with ASD compared to those in low‐risk infants who were not diagnosed with ASD (Lewis et al., 2017). This difference could be explained by the idea that underconnectivity and overconnectivity in topological networks, with age‐dependent regional variation, coexist in preschool‐aged children with ASD and give rise to aberrant connectivity. Another plausible explanation for such inconsistencies is age‐related differences. While previous studies investigated ASD in toddlers aged 1–4 years, our study focused on children aged 3–6 years. Along with these possible interpretations of regional variation, we further speculate that these inconsistencies may be attributable to the varied ASD evaluations used. Previous research used either the ADOS or CARS clinical scores for evaluation, whereas both ADOS and CARS were assessed in our study, potentially increasing the accuracy and reliability of results.

### Potential relationship with connectivity in the HG and ITG of individuals with low‐functioning ASD

Of note, the nodal strength of the right HG was associated with increased ASD severity. Furthermore, significantly increased nodal strength and hyper‐connectivity were identified by NBS in the ASD group. The HG has been ignored in topological network studies of ASD using graph theory, with only a limited number of studies examining volume (Prigge et al., 2013) and asymmetry (Knaus et al., 2009; Rojas et al., 2002). More focus should be placed on the role of the HG in early preschool age as an ROI in autism research, since some of the core symptoms of individuals with autism are delayed language and heightened auditory sensitivity.

The transverse gyrus of the HG, the most anterior transverse gyrus on the superior temporal plane, plays a pivotal role in early acoustic processing as the primary auditory cortex (Galaburda & Kemper, 1978; Galaburda & Sanides, 1980). Abnormalities in the early processing of incoming auditory stimuli affect subsequent sensory perception and higher‐order processing, such as speech perception and acquisition (Kuhl, 2004). In a functional MRI study of adults with ASD, increased activity in the HG was observed in the ASD group in response to temporally complex sounds, indicating a sensitive perceptual feature of auditory processing in individuals with ASD (Samson et al., 2011). A longitudinal study of HG growth in individuals with ASD between the ages of 3 and 12 years showed an atypical trajectory of the right HG volume compared to the TDC group, although there was no difference between the ASD and TDC groups at any particular timepoint (Prigge et al., 2013). Consequently, increased connectivity leads to increased nodal strength and may affect the severity of ASD, considering the significant positive correlation between nodal strength and symptoms. We suggest that accelerated connection primarily in the right HG, which is important for early auditory processing, may prematurely hinder developmental opportunities to acquire social skills and higher‐order processing.

Similar to the HG findings, along with the increased nodal strength and hyper‐connectivity identified by NBS, the second finding of a mid‐level, but marginally significant (*r* = 0.5241, *p* = 0.0544) positive relationship between the nodal strength of the ITG and CARS total score suggests that the ITG may reflect the behavioral and clinical symptoms of ASD. The ITG is involved in the ventral streams of visual processing and plays a role in language and cognition through its connection with the rest of the cortical area. The primary functions of the ITG in learning language are visual stimulus processing and visual perception at an early age, which are related to visual object recognition (Herath et al., 2001). In addition, this region is connected with the long‐range fiber of the inferior longitudinal fasciculus and short‐range arcuate fasciculus, which support visual and language comprehension (Yeatman et al., 2012). Considering the importance of the ITG in language acquisition, over‐enhancement of the right network efficiency in our study may partly explain the language disorder and social dysfunction, as well as the occurrence of compensated development for poorly functioning connectivity, in preschool‐aged children with low‐functioning ASD. Cai et al. found an increased gray matter volume in the ITG of patients with low‐functioning ASD compared to in TDC, possibly reflecting delayed language development (Cai et al., 2018). Interestingly, previous studies (Kaldy et al., 2016; Plaisted et al., 1998) reported that infants with ASD had faster visual reactions than controls in a feature‐conjunction task, as early as 2.5 years of age, leading to abnormal visual explorative behavior and exaggerated discrimination. This compensatory mechanism, with a skew towards visual processing, may be prioritized in preschool‐aged children with weak receptive language skills, as they are unable to follow verbal instructions. Our results can be extended in these earlier studies and are supported by neuroimaging analysis using graph theory, suggesting that preschool‐aged children with low‐functioning ASD have an abnormal ITG with aberrant connectivity compared to that in TDC.

However, we were not able to determine whether the aberrant connectivity found in our study was related to ASD symptoms or low IQ. Our results should be interpreted with caution, as this study did not include an IQ‐matched control group (IQ <70, but without ASD). Also, there has been controversy about estimates from IQ scores when assessing the functional abilities in preschool‐aged children with ASD (Alvares et al., 2020), as IQ scores alone do not always correlate with functional ability in preschool‐aged children with ASD. However, the previously mentioned study by Cai et al. found increased gray matter volume in low‐functioning ASD as well as high‐functioning ASD, suggesting that this feature may be implicated in the pathophysiology of ASD across the autism spectrum at different IQ levels (Cai et al., 2018). Another study including children with ASD with lower functional ability and an IQ‐matched control group also observed right amygdala enlargement as a compensatory response in ASD relative to controls was more pronounced between 2 and 4 years of age (Mosconi et al., 2009). With regards to the HG, previous studies have found positive associations in ASD participants as a selective feature (Prigge et al., 2013; Samson et al., 2011), but the relationship with intellectual disability has not been reported, suggesting that the connectivity of the HG may be a unique finding, rather than primarily being affected by IQ level. HG is associated with social attention and language development in preschool‐aged children with ASD. Impairments in these functions may be considered the first behavioral signs between 3 and 6 years of age. However, further studies on individuals with an IQ <70 without ASD as a control group are warranted to differentiate the effects of ASD symptoms and IQ.

### Altered cerebral lateralization in the ASD group

The rightward lateralization of the ITG in the ASD group, relative to the TDC group, may be associated with the aberrant developmental trajectory of right hemispheric dominance in ASD, contributing to communication problems in language processing. This rightward lateralization in the temporal network supports the findings of an earlier brain network study in children with ASD (Conti et al., 2016), where the degree of rightward lateralization in language‐related networks positively correlated with the severity of ASD. An atypical lateralization across the hemisphere in the cerebral cortex and white matter was also reported in a previous longitudinal study, which showed widespread rightward asymmetry in an ASD group between the ages of 2 and 5 years (Fu et al., 2020). These results explain the complexity of the atypical lateralization observed in ASD, where the theory of left hemisphere dysfunction is mostly related to the social and communicative disturbances observed in ASD. However, due to the sample size, we were unable to obtain significant results from the correlation analysis with clinical severity scores. It remains to be determined at which age these atypical patterns of lateralization occur, and particularly, if a reduced structural asymmetry is present at birth, or rather develops over time as a compensatory response to abnormal brain development. A lack of maturation of the left‐lateralized temporal cortex may be an early biological sign of deficient response to speech and language in preschool‐aged children with ASD.

### Limitations

First, the number of subjects was insufficient; thus, the results should be carefully interpreted. However, it should be considered that data collection from a large neuroimaging cohort of very young children is extremely difficult. Second, DTI and deterministic tractography have inherent limitations, including the issue of crossing fibers. Other tracking methods, including probabilistic tractography (Behrens et al., 2007) should be considered in future research.

### Conclusion

Our findings highlight the potential of graph theory‐based network characteristics and underlying structural networks in preschool‐aged children with low‐functioning ASD. Furthermore, increased connectivity in the right HG and ITG in children with ASD may reflect impaired social communication and disrupted interhemispheric connections, supporting the theory of atypical development of the right hemisphere at an early age in preschool‐aged children with low‐functioning ASD. These findings provide a structural foundation for a more comprehensive understanding of neuropathological abnormalities in children with ASD and may facilitate early diagnosis and intervention based on relevant neuroimaging biomarkers of clinical severity for preschool‐aged children with ASD.

## CONFLICT OF INTEREST

The authors declare no competing interests.

## AUTHOR'S CONTRIBUTION

*Conceptualization*: Hyun Ju Lee and Cheol E. Han. *Formal analysis*: Daegyeom Kim and ByeongChang Jeong. *Investigation*: Joo Young Lee, Ja‐Hye Ahn, Johanna Inhyang Kim, Eun Soo Lee, and Hyuna Kim. *Methodology*: Daegyeom Kim, ByeongChang Jeong, and Hyun Ju Lee. *Resources*: Hyun Ju Lee and Johanna Inhyang Kim. *Writing‐original draft*: Daegyeom Kim, Joo Young Lee, and Eun Soo Lee.

## Supporting information


**Supplementary Table 1** Six studies using graph theory to analyze the structural network connectivity in preschool‐aged children with ASD

**Supplementary Table 2** Differences in global measures between ASD children and TDC

**Supplementary Table 3** Correlation between the global measures and symptoms in the ASD group

**Supplementary Table 4** Asymmetry difference of nodal strength between ASD children and TDC, and their correlations with ASD symptoms
