# Supplementary material for: Overconnectivity of the right Heschl's and inferior temporal gyrus correlates with symptom severity in preschoolers with autism spectrum disorder
Source: Autism Res. 2021 Sep 16;14(11):2314–29. doi: 10.1002/aur.2609 (PMC9292809; doi:10.1002/aur.2609)
Supplement: Supplementary file 1 — Supplementary Table 1 Six studies using graph theory to analyze the structural network connectivity in preschool‐aged children with ASD [file AUR-14-2314-s004.docx]

**Supplementary Table 1 Six studies using graph theory to analyze the structural network connectivity in preschool-aged children with ASD**

| **Study** | **Demographic characteristics** | | | | | | | | |  |  |  |  |
| --- | --- | --- | --- | --- | --- | --- | --- | --- | --- | --- | --- | --- | --- |
|  | **ASD** | | | |  | **TDC** | | | |  |  |  |  |
|  | **N** | **Males** | **Age range**  **(years)** | **IQ** |  | **N** | **Males** | **Age range**  **(years)** | **IQ** | **Diagnostic tools** | **Tesla** | **Number of directions** | **Main findings** |
| Billeci et al., 2019 | 16 | 16 | 1.5‒5.2 | 83.7 |  | NA | NA | NA | NA | DSM-V, ADOS | 1.5 | 30 | Local network measures (local efficiency, clustering coefficient, betweenness centrality) was positively correlated with autism severity |
| Carpenter et al., 2019 | 19 | 17 | 2‒6 | NA |  | NA | NA | NA | NA | DSM-V, ADOS | 3.0 | 25 | Autism severity was correlated with increased connectivity between frontal pole and the globus pallidus |
| Qian et al., 2018 | 37 | 32 | 2‒5 | NA |  | 27 | 21 | 2‒5 | NA | DSM-IV, ADI-R, CARS | 3.0 | 30 | Nodal efficiency of the left precuneus was positively correlated with autism severity |
| Qin et al., 2018 | 39 | 32 | 2‒6 | NA | 1 | 19 | 13 | 2‒6 | NA | DSM-IV, CARS | 3.0 | NA | Significant positive correlation between nodal efficiency of the left precuneus and the CARS in autism severity |
| Li et al., 2018 | 21 | 14 | 4‒6 | 89‒100 |  | 21 | 11 | 4‒6 | 90-110 | DSM-V, ABC, CARS | 3.0 | 24 | Nodal efficiency in left pallidum and right caudate nucleus was positively correlated with in autism severity |
| Lewis et al., 2014 | 31 | 24 | 2.07 | NA |  | NA | NA | NA | NA | ADI-R, ADOS | 3.0 | 25 | Nodal efficiency in left temporal lobe was negatively correlated with autism severity |

Abbreviations: ASD, autism spectrum disorder; TDC, typically developing children; IQ, intelligence quotient; DSM, diagnostic and statistical manual of mental disorders; ADOS, autism diagnostic observation schedule; ADI-R, autism diagnostic interview-revised; CARS, childhood autism rating scale;, ABC, autism behavior checklist; NA, not available
