# Supplementary material for: Overconnectivity of the right Heschl's and inferior temporal gyrus correlates with symptom severity in preschoolers with autism spectrum disorder
Source: Autism Res. 2021 Sep 16;14(11):2314–29. doi: 10.1002/aur.2609 (PMC9292809; doi:10.1002/aur.2609)
Supplement: Supplementary file 2 — Supplementary Table 2 Differences in global measures between ASD children and TDC [file AUR-14-2314-s002.docx]

**Supplementary Table 2. Differences in global measures between ASD children and TDC**

| Global measures | TDC | ASD | p-value^†^ |
| --- | --- | --- | --- |
| Total strength | 24983 ± 2589 | 25774 ± 2377 | 0.0849 |
| Edge density | 0.1732 ± 0.0072 | 0.1738 ± 0.0094 | 0.5861 |
| Clustering coefficient | 13.0017 ± 1.1801 | 13.2842 ± 1.0370 | 0.2166 |
| Characteristic path length | 0.0469 ± 0.0055 | 0.0458 ± 0.0045 | 0.1893 |
| Local efficiency | 23.8934 ± 2.1890 | 24.3602 ± 1.7312 | 0.1970 |
| Global efficiency | 29.8573 ± 3.3101 | 30.7784 ± 2.7810 | 0.1166 |
| Small-worldness | 282.9291 ± 52.8701 | 294.1965 ± 43.8113 | 0.1792 |

Data are presented as the mean ± standard deviation.

^†^ P-values from the permutation-based ANCOVA, controlling for GA, sex and age at imaging

Abbreviation: ASD, autism spectrum disorder; TDC, typically developing children
