# Supplementary material for: Overconnectivity of the right Heschl's and inferior temporal gyrus correlates with symptom severity in preschoolers with autism spectrum disorder
Source: Autism Res. 2021 Sep 16;14(11):2314–29. doi: 10.1002/aur.2609 (PMC9292809; doi:10.1002/aur.2609)
Supplement: Supplementary file 3 — Supplementary Table 3 Correlation between the global measures and symptoms in the ASD group [file AUR-14-2314-s003.docx]

**Supplementary Table 3. Correlation between the global measures and symptoms in the ASD group**

| Global measures | CARS | | ADOS (SA) | | ADOS (RRB) | | ADOS (TOTAL) | | |
| --- | --- | --- | --- | --- | --- | --- | --- | --- | --- |
|  | r-value | p-value | r-value | p-value | r-value | p-value | r-value | p-value | |
| Total strength | 0.3397 | 0.2308 | -0.3725 | 0.3803 | 0.3845 | 0.2419 | -0.0540 | 0.9997 |  |
| Edge density | 0.4867 | 0.1771 | -0.3030 | 0.3880 | 0.5697 | 0.0952 | 0.0966 | 0.9997 |  |
| Clustering coefficient | 0.0942 | 0.6845 | -0.0200 | 0.9372 | 0.1274 | 0.6271 | 0.0555 | 0.9997 |  |
| Characteristic path length | -0.3975 | 0.1897 | 0.3438 | 0.3803 | -0.3690 | 0.2419 | 0.0421 | 0.9997 |  |
| Local efficiency | 0.1483 | 0.6080 | -0.1309 | 0.7054 | 0.1229 | 0.6271 | -0.0257 | 0.9997 |  |
| Global efficiency | 0.3890 | 0.1897 | -0.3434 | 0.3803 | 0.3635 | 0.2419 | -0.0448 | 0.9997 |  |
| Small-worldness | 0.2890 | 0.2855 | -0.2262 | 0.5135 | 0.2936 | 0.3319 | 0.0001 | 0.9997 |  |

Data are presented as the partial correlation coefficient and FDR-adjusted p-value, controlling for gestational age, sex and age at imaging.

Abbreviations: ASD, autism spectrum disorder; CARS, childhood autism rating scale; ADOS, autism diagnostic observation schedule; SA, social affective; RRB, restrictive repetitive behavior
