# Supplementary material for: Overconnectivity of the right Heschl's and inferior temporal gyrus correlates with symptom severity in preschoolers with autism spectrum disorder
Source: Autism Res. 2021 Sep 16;14(11):2314–29. doi: 10.1002/aur.2609 (PMC9292809; doi:10.1002/aur.2609)
Supplement: Supplementary file 4 — Supplementary Table 4 Asymmetry difference of nodal strength between ASD children and TDC, and their correlations with ASD symptoms [file AUR-14-2314-s001.docx]

**Supplementary Table 4. Asymmetry difference of nodal strength between ASD children and TDC, and their correlations with ASD symptoms**

|  | Group difference | | | Correlation with ASD symptoms | | |
| --- | --- | --- | --- | --- | --- | --- |
| Nodes | TDC | ASD | p-value^†^ | Scores | r-value^‡^ | p-value^‡^ |
| Heschl’s gyrus | 0.1067 [0.0550‒0.1613] | 0.1774 [0.0863‒0.2548] | 0.0551 | CARS | -0.1277 | 0.5813 |
|  | (p < 0.0001^*^) | (p < 0.0001^*^) | - | ADOS (TOTAL) | 0.3653 | 0.1361 |
|  | - | - | - | ADOS (SA) | 0.4491 | 0.0615 |
|  | - | - | - | ADOS (RRB) | 0.0853 | 0.7366 |
|  |  |  |  |  |  |  |
| Inferior temporal gyrus | -0.0540 [-0.1028‒0.0012] | 0.0226 [-0.0395‒0.0696] | 0.0072 | CARS | 0.3320 | 0.1415 |
|  | (p = 0.0227^*^) | (p = 0.5716^*^) | - | ADOS (TOTAL) | 0.2084 | 0.4066 |
|  | - | - | - | ADOS (SA) | 0.1573 | 0.5332 |
|  | - | - | - | ADOS (RRB) | 0.1770 | 0.4823 |

Data are presented as the median and interquartile range of asymmetry index (AI)

^*^ P-values from Wilcoxon signed-rank tests.

^†^ P-values from the rank-sum test for group comparisons

^‡^ Partial correlation coefficient and its P-value, controlling for GA, age and gender at imaging

Abbreviations: ASD, autism spectrum disorder; TDC, typically developing children; ADOS, autism diagnostic observation schedule; SA, social affective; RRB, restrictive repetitive behavior; CARS, childhood autism rating scale
